# Histiocytes set on the heart: cardiac complications of Erdheim–Chester disease

**DOI:** 10.1093/eurheartj/ehac769

**Published:** 2023-02-18

**Authors:** Matthew Collin

**Affiliations:** Translational and Clinical Research Institute, Newcastle University, Framlington Place, Newcastle upon Tyne NE2 4HH, UK

## Abstract

**Graphical Abstract:**
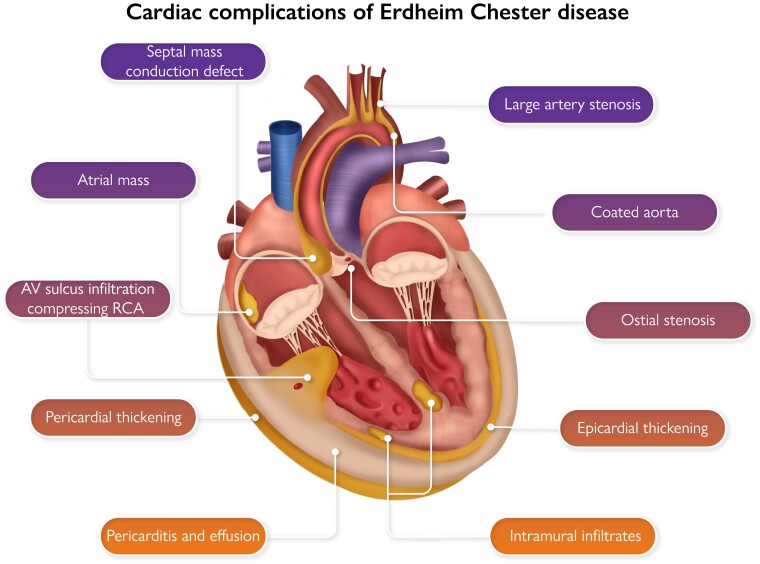
Sites of cardiac involvement by Erdheim–Chester disease.

Sites of cardiac involvement by Erdheim–Chester disease.

**This editorial refers to ‘Prevalence, patterns, and outcomes of cardiac involvement in Erdheim–Chester disease’, by L.-D. Azoulay *et al*., https://doi.org/10.1093/eurheartj/ehac741.**

Most patients with Erdheim–Chester disease (ECD) complain that doctors have never heard of their condition. Or if they have, it remains buried in the recesses of medical school knowledge as the smart reply to almost any clinical mystery—one of the great disease mimics. It is true that ECD can affect almost any organ including the central nervous system (CNS), bone, skin, lung, kidney, and vasculature, and it should indeed be on many differentials.^1,2^ In this issue of the *European Heart Journal*, Azoulay and colleagues focus on the heart.^3^

ECD is a rare disease but not as rare as textbooks suggest. The true incidence is ∼1 per million, affecting adults between 50 and 70 years of age with a 2:1 male predominance.^4,5^ ECD affects so many organs because it is a histiocytic neoplasm; a clonal expansion of macrophages caused by a mutation in the bone marrow.^6,7^ Macrophages are found in almost all tissues including the myocardium, vasculature, serosal surfaces, and adipose and interstitial tissues. ECD is a form of clonal haematopoiesis but not simply an age-related clonal haematopoiesis of indeterminate potential—also known as ‘CHIP’.^8^ ECD has very definite disease-causing potential in the cardiovascular system, depositing neoplastic activated macrophags in multiple locations—a kind of ‘super-CHIP’. Histiocytic neoplasms are also related to myeloproliferative neoplasm such as essential thrombocythaemia and polycythaemia vera—both well known for their thrombotic potential and cardiovascular risk. As macrophages are so intimately involved in inflammation, including atheroma, it can be difficult to identify ECD under the microscope. ECD macrophages tend to eat lipid and become fat-laden and foamy (‘xanthomatous’ in pathology reports) but they can also be found with vigorous mononuclear infiltrates and dense fibrosis like many other conditions. For this reason, tracking down the causative mutation is extremely useful in making the diagnosis.^1,2^ It is also important for treatment since mutations are almost always in the mitogen-activated protein (MAP) kinase pathway (RAS–RAF–MEK–ERK) and can be stopped in their tracks by widely available BRAF and MEK inhibitors—Dabrafenib and Trametinib, for example—designed to treat melanoma. Life-threatening cardiac complications will stabilize and resolve with prompt diagnosis and treatment with inhibitors.^1,2^ The article by Azoulay and colleagues is timely: ECD is no longer a curiosity we can afford to miss but a potentially treatable cause of serious cardiac dysfunction.

The tissue tropism of ECD is incompletely understood, but many of the sites of involvement are adipose-rich tissues such as the bone marrow and peri-nephric regions. Cardiac manifestations of ECD are often underdiagnosed and may include myocardial infiltrates, right atrial inflammatory pseudotumour, infiltration of the atrioventricular sulcus, pericardial thickening, effusion, and tamponade.^3^ Vascular disease is commonly seen with stenosis of the coronary ostia and right coronary artery, and thickening or ‘coating’ of the aorta. Myocardial infiltrates may appear as atypical infarcts, unrelated to vascular territories, and can disrupt conduction and impair ventricular function. They typically show late gadolinium enhancement on cardiac magnetic resonance (MR). Other features that should alert clinicians to the possibility of ECD are large peri-orbital xanthomata (with a normal lipid profile) renovascular disease, coeliac axis insufficiency, and weak or absent pulses often due to short proximal stenoses of large arteries. Classic non-cardiovascular features of ECD include bone pain and sclerosis, diabetes insipidus, retroperitoneal fibrosis, monocytosis, and raised inflammatory markers. Positron emission tomography-computed tomography (PET-CT) is usually diagnostic. Cardiac MR or gated CT are mandatory for full assessment of the heart.

Azoulay and colleagues found that almost half of their patients had cardiac involvement, most commonly infiltration of the right atrioventricular sulcus, seen in three-quarters of those affected. Infiltration of the atria, pseudotumour, and pericardial disease also occurred in more than half of affected patients. Cardiac involvement was significantly associated with coated aorta and with the mutation *BRAF^V600E^* which is often detectable in circulating monocytes and cell-free DNA. Although coronary artery disease is probably more common than in the general population, it was not specifically associated with cardiac involvement within ECD patients. Coronary insufficiency may also result from extrinsic compression by atrial and sulcal inflammation; an unusual finding that should raise suspicion of ECD. Five-year survival was 81% in this relatively young patient group (median age 60) and not significantly linked to freedom from cardiac involvement.

Caring for patients with ECD is by nature a multidisciplinary activity, and engagement with cardiologists is essential to investigate and manage cardiac involvement optimally. A patient-led organization, the ECD Global Alliance, has been critical in raising awareness over the last few years, and has many resources for patients and clinicians interesting in learning more: https://erdheim-chester.org.

## Data Availability

No new data were generated or analysed in support of this research.
